# The Role of Epithelial-to-Mesenchymal Plasticity in Ovarian Cancer Progression and Therapy Resistance

**DOI:** 10.3390/cancers11060838

**Published:** 2019-06-17

**Authors:** Nele Loret, Hannelore Denys, Philippe Tummers, Geert Berx

**Affiliations:** 1 Molecular and Cellular Oncology Laboratory, Department of Biomedical Molecular Biology, Ghent University, Technologiepark 71, 9052 Ghent, Belgium; nele.loret@ugent.be; 2 Cancer Research Institute Ghent (CRIG), 9000 Ghent, Belgium; Hannelore.Denys@UGent.be (H.D.); philippe.tummers@ugent.be (P.T.); 3 Department of Gynaecologic Oncology, Ghent University Hospital, C. Heymanslaan 10, 9000 Ghent, Belgium; 4 VIB Center for Inflammation Research, Technologiepark 71, 9052 Ghent, Belgium; 5 Department of Medical Oncology, Ghent University Hospital, C. Heymanslaan 10, 9000 Ghent, Belgium

**Keywords:** ovarian cancer, epithelial-to-mesenchymal transition (EMT), mesenchymal-to-epithelial transition (MET), peritoneal metastasis, therapy resistance, cancer stem cell (CSC)

## Abstract

Ovarian cancer is the most lethal of all gynecologic malignancies and the eighth leading cause of cancer-related deaths among women worldwide. The main reasons for this poor prognosis are late diagnosis; when the disease is already in an advanced stage, and the frequent development of resistance to current chemotherapeutic regimens. Growing evidence demonstrates that apart from its role in ovarian cancer progression, epithelial-to-mesenchymal transition (EMT) can promote chemotherapy resistance. In this review, we will highlight the contribution of EMT to the distinct steps of ovarian cancer progression. In addition, we will review the different types of ovarian cancer resistance to therapy with particular attention to EMT-mediated mechanisms such as cell fate transitions, enhancement of cancer cell survival, and upregulation of genes related to drug resistance. Preclinical studies of anti-EMT therapies have yielded promising results. However, before anti-EMT therapies can be effectively implemented in clinical trials, more research is needed to elucidate the mechanisms leading to EMT-induced therapy resistance.

## 1. Introduction

In 2018, the worldwide age-standardized incidence of ovarian cancer was 6.6/100,000 [1]. With an overall five-year survival rate of only 38–40%, epithelial ovarian cancer (EOC) is the most lethal gynecologic malignancy [2,3], and the eighth leading cause of cancer-related deaths among women worldwide [1]. This poor prognosis is mainly due to late-stage diagnosis and the frequent development of resistance to current chemotherapy regimens [4,5]. 

According to the WHO (World Health Organization) classification, there are seven main histological subtypes of EOC: high-grade and low-grade serous, mucinous, endometrioid, clear cell, Brenner, seromucinous, and undifferentiated carcinomas [6,7]. The high-grade serous (HGS) subtype, of which the majority (>80%) arise from the fallopian tube epithelium [8], is responsible for the largest number of ovarian cancer deaths [9,10]. The majority of HGS cancers are associated with *TP53* mutations and approximately half of them have aberrant DNA repair via homologous recombination (because of genetic or epigenetic alterations of BRCA1/2 or other DNA repair molecules) [11,12]. On the other hand, low-grade serous carcinomas are mostly characterized by *BRAF*- and *KRAS*-mutations [12]. The typical molecular alterations found in the other subtypes are *KRAS*-, *CDKN2A*- and *TP53*-mutations for the mucinous subtype, *BRAF*-, *PTEN*-, *ARID1A*- and *CTNNB1*-mutations for the endometrioid subtype, and *PIK3CA*- and *ARID1A*-mutations for the clear cell ovarian carcinomas [13]. 

The extent of the disease is classified according to the FIGO (Fédération Internationale de Gynécologie et d’Obstétrique) staging system. In stage I, the tumor is confined to the ovaries or the fallopian tube(s), whereas in stage II the tumor has already extended to the pelvis (below the pelvic brim). However, most ovarian cancers are diagnosed in stage III when the tumor has already spread to the peritoneum outside the pelvis and/or the retroperitoneal lymph nodes. Stage IV is characterized by distant metastases other than peritoneal metastases. [14].

Current first-line treatment for epithelial ovarian cancer consists of complete debulking surgery combined with chemotherapy (carboplatin and paclitaxel). Although most patients initially respond well to primary combined treatment, about 80% relapse within five years [3]. This underscores the need to elucidate the mechanisms driving therapy resistance and to develop therapeutic strategies targeting the resistant relapse-inducing cancer cells. 

## 2. Epithelial-to-Mesenchymal Transition (EMT)

### 2.1. Definition of A Complex Process

EMT is a reversible process by which epithelial cells lose their apical–basal polarity and cell–cell adhesion to become more spindle-shaped mesenchymal cells with increased migratory capacities. During this process, E-cadherin, an important component of adherens junctions, is repressed as well as occludins, claudins, Epcam, α6β4 integrin, and different cytokeratins, which are important for stabilization of desmosomes. Simultaneously, vimentin, fibronectin, neural cadherin (N-cadherin), β1 and β3 integrins, and matrix metalloproteinases (MMPs) are upregulated [15]. These mesenchymal-like cells can revert to their epithelial state, a process called mesenchymal-to-epithelial transition (MET) [15]. Moreover, recent evidence indicates that cancer cells can also be in an intermediate “partial EMT”-state with characteristics of both epithelial and mesenchymal cells [16,17,18]. 

Depending on the context in which EMT occurs, three types of EMT can be distinguished. Type I is important during embryogenesis, type II is observed during wound healing and fibrosis, and type III is associated with cancer progression [19]. Type III EMT drives the tumor-initiating- and metastasizing capacity of cancer cells as well as increased resistance to chemo- and immunotherapy [15]. Moreover, it makes cancer cells more resistant to anoïkis [20]. 

### 2.2. EMT Transcription Factors

EMT is regulated by a complex network of transcription factors that eventually lead to downregulation of epithelial genes and upregulation of mesenchymal genes. The leading EMT transcription factors are the zinc-finger E-box-binding homeobox factors Zeb1 and Zeb2, Snail (SNAI1), Slug (SNAI2), and the basic helix-loop-helix factors Twist1 and Twist2 [15,21,22,23,24,25,26]. Snail and Zeb transcription factors repress E-cadherin by directly binding it [27,28].

## 3. EMT and High-Grade Serous Ovarian Cancer Progression

### 3.1. The Role of EMT in HGS Ovarian Cancer Initiation 

**Figure 1:**
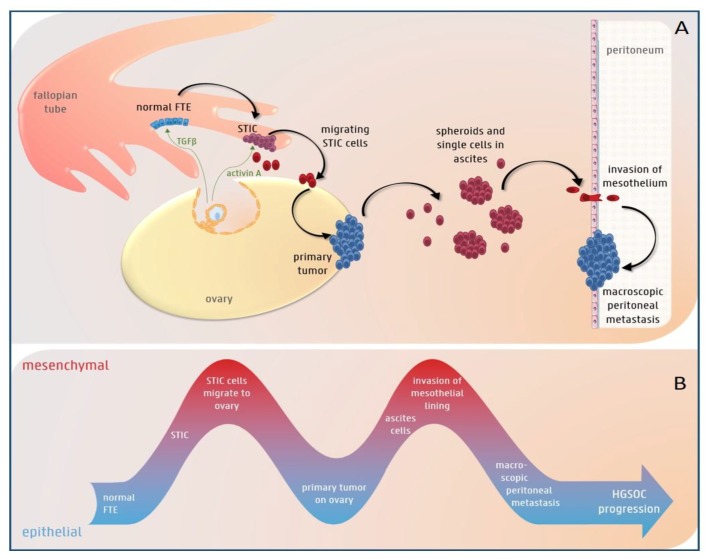
The potential role of epithelial-to-mesenchymal (EMT) plasticity during high-grade serous (HGS) ovarian cancer progression. (**A**) The color of the cells represents their EMT-state (blue = epithelial, purple = spectrum of epithelial/mesenchymal differentiation and red = mesenchymal). TGFβ present in follicular fluid, which is released during ovulation, can induce EMT in the normal fallopian tube epithelium (FTE). This can lead to the development of STIC (serous tubal intraepithelial carcinoma) lesions. Acitivin A, another component of the follicular fluid, stimulates migration of the STIC cells to the ovary, where they undergo mesenchymal-to-epithelial transition (MET) and form a primary tumor. In a later stage, cells exfoliate from the primary tumor and survive as single cells or spheroids in the ascites. Finally, they invade the mesothelium and again undergo MET to form macroscopic peritoneal or omental metastases. (**B**) EMT plasticity with EMT and MET alternately taking place during HGS ovarian cancer progression.

As previously demonstrated in other tumor types, a small subset of “tumor initiating cells” has been identified in ovarian cancer [29,30,31,32,33,34,35,36]. These cells show mesenchymal- and stem cell-features, and are thought to drive tumor initiation [37]. The contribution of EMT to ovarian cancer initiation is indicated by the finding that induction of EMT caused repression of paired box protein 2 (PAX2), a transcription factor that preserves the differentiation state of oviductal epithelial cells. This in turn led to the development of HGS ovarian cancer precursor lesions, so-called secretory cell outgrowths (SCOUTS) and serous tubal intraepithelial carcinomas (STIC) [30]. This EMT and subsequent emergence of HGS ovarian cancer precursor lesions could be initiated by various stimuli, including TGFβ, a potent EMT-inducer present in the follicular fluid, which is released during ovulation [30,38] (Figure 1).

Furthermore, there is growing evidence that loss of breast cancer type 1 susceptibility protein (BRCA1) is associated with EMT and tumor initiation. BRCA1 is a tumor suppressor that plays a role in the repair of double-stranded DNA breaks. Women with germline BRCA1 mutations have an inherited predisposition to breast- and ovarian cancer, with lifetime risks of 60–80% and 40–60%, respectively [39,40,41]. They mainly develop aggressive, high-grade, and dedifferentiated tumor subtypes, such as triple negative breast cancers and HGS ovarian cancers, with loss of differentiation, aggressive behavior, and poor prognosis [41,42]. The link between BRCA1 and EMT in breast cancer has been demonstrated (reviewed in [41]), but so far it has not been investigated in ovarian cancer. In breast cancer, loss of BRCA1 results in dedifferentiation of mammary epithelial cells to a more stem cell like phenotype, with upregulation of CD44 and induction of EMT. Together with the ATPase-dependent chromatin remodeling protein (BRG1), and the Fanconi anemia group D2 protein (FANCD2), BRCA1 forms a complex important for inter-strand DNA crosslink repair and induction of ΔNp63 expression [43]. ΔNp63, a p63 isoform with pleomorphic functions, can prevent EMT in human mammary cells. This indicates that BRCA1-mediated DNA repair is important for maintaining a normal differentiation state and suppressing the development of breast- and ovarian cancer [43,44]. Indeed, reduced BRCA1 expression induced tumor-initiating cells, EMT, and stemness in breast cancer [45]. This association between BRCA1 and key molecules of EMT might explain why patients carrying mutations in BRCA1 develop mainly aggressive and dedifferentiated serous ovarian cancers. Nevertheless, this link still needs to be investigated in ovarian cancer.

### 3.2. EMT Plasticity during HGS Ovarian Cancer Progression

Growing evidence shows an important role for EMT in ovarian cancer metastasis [32,46,47,48]. In the next paragraph, we will highlight the contribution of EMT to the steps of ovarian cancer progression.

Once a STIC lesion has formed, cells exfoliate from the fimbriae and reach the ovarian surface, where they form an invasive carcinoma [49]. The migration of STIC cells to the ovary is an important step in HGS ovarian cancer progression (Figure 1). Notably, oophorectomy in a transgenic mouse model with fallopian tube-derived cancer reduced peritoneal metastases [50]. Indeed, the ovaries secrete many growth factors and hormones, such as TGFβ and activin A, which might attract cancer cells to the ovarian surface. Activin A, a member of the TGFβ superfamily present in the follicular fluid released during ovulation, induced EMT and stimulated migration of fallopian tube epithelium cells and HGS ovarian cancer cells by activation of the non-canonical PI3/AKT and MEK/ERK pathways [51]. This points to a pivotal role for activin A-induced EMT in the early stages of HGS ovarian cancer progression [51].

In the more advanced stages, cancer cells metastasize to the peritoneal cavity. In contrast to most epithelial cancers, ovarian cancer metastasizes mainly via a transcoelomic rather than a hematogenous route [52]. In this type of dissemination, cancer cells are shed from the primary tumor directly into the peritoneal cavity and survive as single cells or multi-cellular spheroids in the malignant intra-abdominal fluid, which is called ascites [53]. When tumor cells exfoliate from the primary tumor into the peritoneal fluid, they are exposed to insufficient cell-matrix interactions. This commonly triggers apoptotic cell death or anoikis in normal cells. EMT, induced by different stimuli such as TGFβ in ascites or by shear stress due to circulating ascites [54], can induce anoikis resistance, leading to survival of ovarian cancer cells in ascites (Figure 1) [55]. Indeed, exfoliated ascitic and metastatic ovarian cancer cells had increased Zeb2- and Akt2-expression compared to in situ lesions [56] and weaker E-cadherin expression compared to the primary tumor [57]. Thereafter, the circulating ascites disseminates the cancer cells into the abdominal cavity. Once the spheroids reach a secondary site, EMT induces upregulation of the fibronectin receptor, α5β1 integrin, which mediates their attachment to the mesothelial lining [47,58]. Indeed, spheroids with a mesenchymal or intermediate mesenchymal phenotype were more resistant to anoikis and invaded more easily than epithelial spheroids into the mesothelium [59]. After invading into the submesothelial matrix, cancer cells undergo the reverse process, MET, to grow out and form macroscopic peritoneal and omental metastases (Figure 1) [60,61,62,63]. These metastases are characterized by an E-cadherin expression level equal to or lower than that of the primary tumor [61,64]. Aside from this transcoelomic dissemination route, recent evidence points to the co-existence of a hematogenous dissemination route to the omentum via activation of the ErbB3-neuregulin1 (NRG1) pathway [65].

Growing evidence indicates that cells in a partial EMT-state are even more aggressive than cells with a complete mesenchymal phenotype, indicating that partial EMT might drive ascites formation and development of peritoneal metastases in ovarian cancer. Indeed, ovarian cancer cells in a hybrid epithelial/mesenchymal (E/M) state showed cancer stem cell (CSC) features and could drive tumor growth in vivo [66]. Moreover, ascites-associated multi-cellular aggregates with a “hybrid cadherin” phenotype, in which the cells are positive for both E-cadherin and N-cadherin, were more invasive than pure epithelial multi-cellular aggregates [67]. In addition, the intermediate mesenchymal subgroup of ovarian cancer cell lines, but not the mesenchymal subgroup, exhibited the strongest anoikis resistance, as well as migratory and invasive capacities in vitro and displayed high expression levels of Zeb1 [68]. These findings are supported by immunohistochemical stainings of human ovarian cancer samples, which showed that the serous subtype in particular is often double-positive for E-cadherin and vimentin [69].

Collectively, these findings provide evidence for the contribution of EMT plasticity to ovarian cancer progression. Ovarian cancer cells can alternately undergo EMT, possibly as a spectrum of states with both epithelial and mesenchymal differentiation, and MET at different stages of HGS ovarian cancer progression.

### 3.3. The Role of EMT in Low-grade Serous Ovarian Cancer Initiation and Progression

In contrast to HGS ovarian cancers, low-grade serous cancers are believed to arise in the ovary from serous cystadenomas. These evolve into non-invasive serous borderline tumors and then slowly and gradually into invasive low-grade serous cancers [70]. Although the oncogenic role of EMT in this subtype has been studied less extensively, there is some evidence that EMT might contribute to progression of low-grade serous ovarian cancers. Indeed, these tumors had elevated expression of EMT transcription factors such as Snail, Slug, Twist, Zeb, ZNF143, and ZNF281 [71,72]. Interestingly, ovarian surface epithelium (OSE) cells are mesothelial cells that can switch between epithelial and mesenchymal differentiation states, which is important for repairing the ovarian surface after ovulation [73]. Furthermore, EMT might contribute to the progression of non-invasive serous borderline tumors to invasive low-grade serous cancers. Indeed, knockdown of p53 stimulated invasion of serous borderline ovarian tumor cells in vitro by PI3K/Akt-mediated downregulation of E-cadherin [74].

## 4. EMT and Chemotherapy Resistance

The first-line chemotherapy treatment for patients with EOC consists of carboplatin combined with paclitaxel. Platinum agents such as cisplatin and carboplatin (a less nephrotoxic and neurotoxic cisplatin derivative [75]) form DNA cross-links and platinum adducts between DNA and proteins, which causes DNA damage and subsequent cell death [76,77]. Paclitaxel induces cell death by binding the β-tubulin subunit of microtubuli, thus causing microtubule dysfunction and subsequent cell cycle arrest [78].

Working on distinct molecular mechanisms, carboplatin, and paclitaxel act synergistically and eliminate most tumor cells during the initial treatment phase. However, 80% of ovarian cancer patients relapse within five years [3]. This emphasizes the need for deeper insight into the molecular mechanisms leading to therapy resistance.

**Figure 2:**
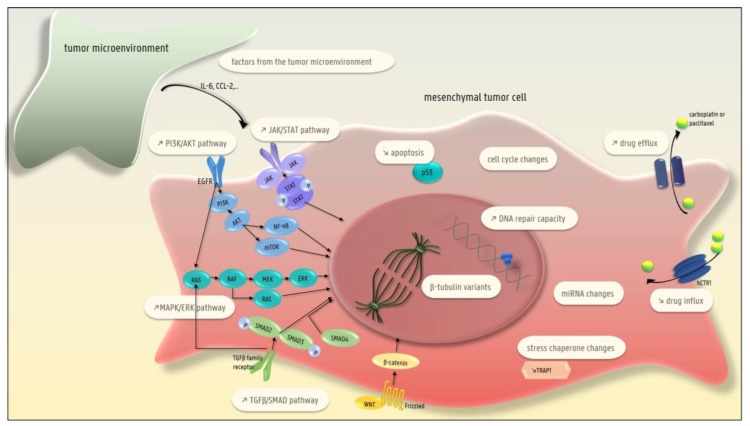
Different EMT-driven mechanisms leading to carboplatin and/or paclitaxel resistance in ovarian cancer cells: the presence of β-tubulin variants; lower drug uptake; higher drug efflux; increased DNA repair capacity; decreased apoptosis; changes in the cell cycle; changes in miRNA; and changes in different pathways (MAPK/ERK, TGFβ-SMAD, JAK/STAT, PI3K-AKT-NFκB). In addition, factors secreted by the microenvironment can induce EMT-related therapy resistance.

Several in vitro and in vivo studies show that cancer cells that are resistant to carboplatin and/or paclitaxel acquire a mesenchymal phenotype, which points to EMT as a driver of resistance to therapy [79,80,81,82,83]. In the following paragraphs, we will discuss the different EMT-driven mechanisms that can lead to chemoresistance (Figure 2).

### 4.1. The Presence of β-Tubulin Variants (Taxane-Specific Resistance)

Six isotypes of β-tubulin have been described, all of which display tissue-specific distribution patterns. Class III β-tubulin (TUBB3) is expressed during early neural differentiation and is normally not present in epithelial cells. Abnormal expression of TUBB3 in ovarian cancer cells induces instability and/or slower polymerization of microtubules which leads to paclitaxel resistance [84]. MiR-200c, a repressor of the EMT-transcription factors Zeb1 and Zeb2, interacted directly with TUBB3 transcripts thereby inhibiting translation. This explains how the miR-200c family can induce sensitivity to paclitaxel [85]. Moreover, short-term treatment of OVCA 433 and HEY ovarian cancer cell lines with paclitaxel promoted EMT and increased the expression of the more paclitaxel-resistant β-tubulin isotypes III and IV [29].

### 4.2. Lower Drug Uptake

Platinum agents enter cells primarily by active transport by the copper transporter 1 (CTR1) [86]. Recent evidence indicates a link between EMT and lower drug uptake. Indeed, more mesenchymal ovarian cancer cell lines that were platinum-resistant expressed lower levels of the copper transporter 1 (CTR1) than their platinum-sensitive and more epithelial counterparts [87]. In line with this, FOXM1 (a member of the forkhead transcription factor family) mediated platinum resistance in human embryonic kidney cells via activation of the WNT/β-catenin pathway, which induces EMT, and inhibition of the human copper transporter 1 (hCTR1), which results in lower drug uptake [88].

### 4.3. Higher Efflux of the Drug

EMT transcription factors can induce the expression of ATP-binding cassette (ABC) transporters. These drug efflux pumps are associated with multidrug resistance, and their promoters contain different binding sites for EMT transcription factors. For example, Twist can directly bind to the E-box elements of ABC transporters [89]. Indeed, P-glycoprotein, a well-known drug efflux pump, was expressed at higher levels in more mesenchymal and therapy-resistant ovarian cancer cell lines, than in more epithelial and chemo-sensitive cell lines [90]. In addition, nidogen-1 (NID-1), which induced EMT and platinum resistance in OVCAR3 and HEY cell lines by stimulating the ERK/MAPK pathway, was associated with upregulation of the drug efflux transporters MDR1 and ABCG2 [91]. The correlation between platinum-resistance, NID1 and stronger expression of MDR1 and ABCG2 was confirmed by whole transcriptome profiling on 28 HGS ovarian cancer tissues [92]. Another example is the sequestration of platinum agents (with consequent lower intracellular platinum concentrations) by the copper-transporting P-type ATPase pump, ATP7B. Downregulation of ATP7B expression by miR-15a and miR-16, two EMT-inhibitors, led to increased cisplatin sensitivity in vitro and in vivo [93,94]. Furthermore, in the mesenchymal OVCAR-8R cells, platinum resistance was mediated by sequestration of platinum by methallothioneins [81].

Furthermore, mounting evidence shows that EMT is a driver of stemness (reviewed in [62]). In analogy with normal stem cells, cancer stem cells protect themselves against chemical mutagens for example chemotherapy, by high expression levels of drug efflux pumps such as ATP-binding cassette transporters, resulting in therapy resistance. In line with this expression of Oct4A, a transcription factor that regulates self-renewal and pluripotency in embryonic stem cells, correlated with CSCs and promoted chemoresistance by upregulating cytoskeletal and extracellular matrix-associated proteins, as well as upregulating ATP-binding cassette sub-family E-member in several ovarian cancer cell lines [62,95,96].

### 4.4. Higher DNA Repair Capacity

EMT can activate enhanced repair of carboplatin-induced DNA damage, counteracting the toxic effect of this chemotherapeutic agent. This is illustrated by sirtuin 6 (SIRT6), an NAD-dependent protein deacetylase, which induced β-catenin mediated EMT and increased DNA repair capacity by activating the DNA repair enzyme, poly ADP-ribose polymerase (PARP). Nuclear SIRT6 expression has been associated with platinum resistance and shorter overall survival in 75 serous ovarian cancer patients [97]. In addition, short-term treatment of OVCA 433 and HEY cell lines with platinum agents induced an EMT-phenotype and upregulated the DNA-excision repair protein ERCC1 (excision repair cross-complementation group 1) [29]. Finally, in an orthotopic ovarian cancer mouse model, miR-506 inhibited EMT and improved the response to cisplatin and olaparib by direct control of RAD51, a double-strand DNA repair gene [98].

### 4.5. Decreased Apoptosis

Snail and Slug induce chemoresistance by inhibiting p53-mediated apoptosis. Both transcription factors confer resistance to p53-mediated apoptosis by inhibiting different pro-apoptotic molecules, such as PUMA/BBC3, ATM, and PTEN [99]. On the other hand, both wild-type TP53 and PUMA are associated with sensitivity to platinum agents in ovarian cancer [100]. Furthermore, Snail exerts an anti-apoptotic effect by stimulating AKT activation (which inhibits p53-mediated apoptosis), slowing down cell-cycle continuation and increasing the expression of Bcl-XL, a pro-survival protein [101]. Indeed, endothelin (ET-1) induced Snail-mediated EMT and chemoresistance in EOC cell lines by activating the PI3-K/Akt pathway and inhibiting apoptosis via a bcl-2 dependent mechanism [102]. Moreover, platinum-resistant OVCAR-8 spheroids were more mesenchymal, and showed increased expression of the apoptosis inhibitors BIRC3 and BCL2L1 compared to platinum sensitive OVCAR-8 spheroids [81].

### 4.6. Changes in the MAPK/ERK Pathway

The MAPK/ERK pathway (also known as the Ras-Raf-MEK-ERK pathway) is known to induce EMT and is also associated with platinum resistance [15,19]. This is exemplified by FOXC2, a member of the forkhead box (FOX) transcription family, which induced resistance to platinum-based chemotherapy in ovarian cancer cells by activating the AKT and MAPK pathways [103]. Another example is nidogen-1 (NID1), which promoted EMT and platinum resistance in OVCAR3 and HEY cells by stimulating the ERK/MAPK pathway [91]. Moreover, inhibiting ERK2 activation with a selective MEK inhibitor decreased platinum-induced EMT and platinum resistance in OVCA 433 cells [104]. In line with this, treating platinum-resistant SKOV3 cells with a MEK inhibitor inhibited ERK phosphorylation and EMT induction and decreased platinum resistance [105].

### 4.7. Changes in EGFR Signaling

The EGFR pathway is activated in more than 70% of ovarian cancer patients and its activation is correlated with platinum resistance and poor prognosis [106].

A link between EMT, EGFR signaling, and therapy resistance has been demonstrated by different authors [107,108,109]. A first example is that of TGFβ1, a potent EMT inducer, which induced the expression of the sialyltransferase ST3GAL. Sialylation of the EGFR by ST3GAL increased EGFR expression and activated EMT, which promoted paclitaxel resistance in vitro and in vivo [108]. In addition, pyruvate dehydrogenase kinase 1 (PDK1) led to EMT and platinum resistance through phosphorylation of EGFR. Erlotinib, an inhibitor of EGFR tyrosine kinase activity, reversed this PDK1-induced platinum resistance in vitro, and silencing PDK1 in vivo also decreased platinum resistance. The authors suggested that PDK1-mediated signaling between the Warburg effect and the EGFR pathway induced chemotherapy resistance [109]. Finally, amphiregulin (AREG), which is inhibited by miR-34c-5p, induced EMT, CSC-like features and resistance to docetaxel and carboplatin in vitro through activation of the AREG-EGFR-ERK pathway. The authors also showed an inverse correlation between AREG expression and overall survival in 65 ovarian cancer patients [107].

Despite the contribution of EGFR-mediated EMT to therapy resistance, a recent Cochrane meta-analysis demonstrated little or no survival benefit from anti-EGFR treatment in ovarian cancer patients [110].

### 4.8. Changes in the TGFβ-SMAD Pathway

The TGFβ signaling pathway is a dominant driver of EMT [111]. Mounting evidence also links this pathway to therapy resistance. This is exemplified by SMAD3, which induces the transcription of serine-threonine tyrosine kinase 1 (STYK1) by directly binding to its promoter. This binding promoted EMT and resistance to paclitaxel, both in vitro and in vivo [112]. Apart from TGFβ, other ligands can activate the TGFβ pathway, such as BMP9, a member of the TGFβ superfamily. BMP9 induced platinum resistance by activating EMT in HO8910 and SKOV3 cell lines [113]. Furthermore, tumor-derived TGFβ stimulated TGFβ secretion by mesothelial cells with subsequent phosphorylation of Smad2 and induction of Zeb1. Indeed, Zeb1 expression was correlated with shorter overall survival in 40 primary EOC samples, and silencing Zeb1 restored sensitivity to paclitaxel in vitro and in vivo [114].

### 4.9. Changes in the PI3-K/AKT/NF-κB and JAK/STAT Pathways

The PI3-AKT/AKT pathway is activated in 40% of ovarian cancers, and this activation is correlated with a poor prognosis [115,116]. Growing evidence shows that EMT mediated by the PI-3K/AKT/NF-κB- and JAK/STAT-pathways can induce chemotherapy resistance, as has been shown in the following publications. As mentioned previously, FOXC2 could mediate platinum resistance in ovarian cancer cells through activation of the AKT and MAPK pathways [103]. A second example is lysyl oxidase (LOX), an enzyme that catalyzes the formation of cross-links between collagen and elastin in the extracellular matrix. Nuclear expression of LOX was associated with increased chemotherapy resistance, shorter progression-free survival and shorter overall survival in 70 advanced HGS ovarian cancer patients. The authors suggested that LOX activated EMT by inducing SLUG and TWIST1 and caused chemotherapy resistance through activation of the PI3K/AKT pathway [117]. Finally, the hematopoietic PBX interacting protein (HPIP) induced EMT and cisplatin resistance by activating the PI3-K/AKT pathway in OAW42 ovarian cancer cells [118].

The importance of the JAK/STAT-pathway in EMT-mediated chemoresistance is illustrated by resistin, a macrophage-derived cytokine associated with obesity and insulin resistance. This cytokine induced platinum resistance, EMT, and stemness in vitro and in vivo by binding Toll-like receptor-4 (TLR4), and by stimulating the NF-κB-STAT3 pathway [119]. Moreover, STAT3 expression was associated with platinum resistance in EOC cells [120].

### 4.10. Changes in the Cell Cycle

Changes in cyclins and cyclin-dependent kinases, which are key regulators of the cell cycle, can also influence the response to chemotherapy. EMT can induce chemoresistance by interfering with the cell cycle, as exemplified by the effects of sperm-associated antigen 9 (SPAG9). Downregulation of SPAG9 in A10- and SKOV-3 ovarian cancer cell lines increased their sensitivity to platinum-based chemotherapy via induction of MET transition and upregulation of the cyclin-dependent kinase inhibitor protein p21, leading to reduced proliferation and cell cycle arrest [121]. In line with this, reduced proliferation mediated by p21, which drives G1 growth arrest, led to resistance to taxanes in eight ovarian cancer cell lines that showed an EMT-signature. Indeed, it is well known that proliferating cells respond better than quiescent cells to taxanes [122].

Mutated TP53 induced Notch3 and subsequently the expression of cyclin G1 (CCNG1), thereby promoting EMT and cisplatin resistance. Conversely, silencing CCNG1 resulted in a more epithelial-like phenotype with weaker expression of CDH2, Snail and Slug, and increased sensitivity to cisplatin in HO8910 and A2780 ovarian cancer cell lines. Moreover, an inverse correlation has been shown between the expression of CCNG1 in HGSOC tissues on the one hand and overall survival and progression-free survival on the other [123].

### 4.11. Changes in Micro-RNAs

The role of micro-RNAs in ovarian cancer and their influence on chemoresistance has been reviewed in several recent papers [124,125,126]. Micro-RNAs can regulate EMT and chemotherapy resistance by interacting with different transcripts, leading to their degradation and consequently preventing their translation. Indeed, a recent paper described nine microRNAs associated with platinum resistance in ovarian cancer, three of which are direct EMT regulators: miR-152, miR-27b, and miR-496 [127].

The best-known EMT-repressing micro-RNA family is the miR-200 family. Cisplatin can directly bind to pre-miR-200b, thereby inhibiting its processing into mature miRNA. In breast cancer cells, decreased expression of miR-200b promoted EMT and subsequent cisplatin resistance by directly inhibiting Zeb1 and Zeb2 [128,129].

Another example is miR-1294, a micro-RNA that enhanced cisplatin sensitivity and induced MET in vitro by directly inhibiting the anti-apoptotic gene insulin-like growth factor 1 receptor (IGF1R), leading to decreased expression of mTOR, AKT, and ErbB. Indeed miR-1294 expression was lower in samples from patients with platinum-resistant ovarian cancer compared to samples from patients with platinum sensitive disease [130].

It has been suggested that miR-363 directly inhibits Snail-induced EMT and thereby ameliorates platinum resistance in vivo. Furthermore miR-363 expression was lower in patients with platinum- resistant EOC compared to platinum sensitive patients [131].

MiR-186 expression suppressed Twist1-induced EMT, and increased sensitivity to platinum-based chemotherapy in vitro and in vivo. Lower miR-186 expression was correlated with therapy resistance and poor survival in serous ovarian cancer samples from patients with FIGO stage IIIC or IV [83].

Whereas the above-mentioned micro-RNAs suppress EMT and therapy resistance, some micro-RNAs such as miR-20a, do the opposite. Indeed, miR-20a induced EMT and cisplatin resistance in OVCAR3 cells [132]. Furthermore, overexpression of miR-181a induced EMT and paclitaxel resistance in SKOV3 cells through upregulation of P-glycoprotein [133]. In addition, miR-216a was shown to induce EMT in liver cancer [134] and was associated with platinum resistance in SKOV3 cells [135]. Moreover, cancer-associated fibroblasts and cancer-associated adipocytes can secrete exosomes containing miR-21, an EMT-inducing micro-RNA [136], which has been shown to induce paclitaxel resistance in OVCA432 and SKOV3 cells [137].

### 4.12. Changes in Stress Chaperones

Stress chaperones bind to client proteins to protect them against irreversible aggregation in response to oxidative, acid and heat stress [138]. Several findings point to a link between stress chaperones, EMT and chemoresistance. The chaperone tumor necrosis factor-associated protein 1 (TRAP1) induces oxidative phosphorylation, with subsequent secretion of cytokines and remodeling of gene expression. Reduced TRAP1 expression caused cisplatin resistance by reducing the inhibition of p70S6K, a kinase that induces Snail (with subsequent E-cadherin repression) and that is often activated in ovarian cancer [139,140,141]. Another example is mortalin, a stress chaperone that induces EMT in breast cancer cells [142]. Silencing mortalin in ovarian cancer cell lines increased platinum sensitivity [143].

### 4.13. Factors Produced by the Tumor Microenvironment

The tumor microenvironment consists of different types of fibroblasts such as cancer-associated fibroblasts (CAFs), as well as immune cells, endothelial cells, and tissue specific and bone marrow-derived mesenchymal stem cells secreting different growth factors and ECM components [144]. This tumor microenvironment can promote ovarian cancer progression and appears to be an attractive therapeutic target [145,146]. Moreover, it is known that the tumor microenvironment can contribute to chemoresistance (reviewed in [147,148]). Indeed, CAFs can promote EMT and therapy resistance in ovarian cancer by secreting IL-6, which activates the JAK/STAT pathway and induces C/EBP transcription factors [149,150] (reviewed in [151]). Furthermore carcinoma-associated mesenchymal stem cells induced platinum resistance in EOC cells and in a mouse model, via a Hedgehog–BMP4 signaling loop [152]. In addition, adipose-derived mesenchymal stem cells induced platinum resistance in EOC cells, probably by decreasing intracellular platinum accumulation [153]. Another example is chemokine (C-C motif) ligand 2 (CCL2), which attracts monocytes and macrophages to the tumor microenvironment. The attracted cells are named tumor-associated macrophages (TAMs). Inhibiting mouse stromal CCL2, an inducer of chemotherapy resistance, increased the therapeutic effect of carboplatin-paclitaxel [154].

Together, these findings highlight the key role of the tumor microenvironment in the development of therapy resistance and point to novel therapeutic targets for ovarian cancers that are refractory to therapy.

## 5. EMT and Immunotherapy Resistance

Ovarian cancer is an immunogenic tumor and a strong intratumoral CD8^+^ T-cell infiltration has been associated with improved overall survival [155]. Multiple pre-clinical and clinical studies are currently evaluating the effect of different immunotherapeutic regimens on ovarian cancer patients [156,157]. Recent reports indicate that EMT can also drive resistance to immunotherapy in several types of cancer [158,159,160,161]. Cells that undergo EMT can upregulate programmed cell death-ligand 1 (PD-L1), which inhibits the cytotoxicity of T cells by binding to their programmed cell death protein 1 (PD-1) receptor [62]. In breast cancer cells, induction of PD-L1, which was suppressed by miR-200, might be caused indirectly by ZEB1, by decreasing miR-200 expression [160]. In breast cancer, PD-L1 expression was induced upon PI3/AKT-mediated EMT [162]. Furthermore, by secreting thrombospondin-1 (TSP-1), cells that have undergone EMT can promote the generation of regulatory T cells (T regs), which suppress the cytotoxic T cell function [62]. In gastric cancer cells, it was shown that PD-L1 expression was induced via the STAT3 and mTOR pathways [163], both of which are well-known EMT-activating pathways [164,165]. Notably, anti-VEGF treatment suppressed PD-L1 expression and EMT via decreased STAT3 phosphorylation in cisplatin resistant ovarian cancer cells. Moreover, anti-VEGF and anti-PD-L1 agents acted synergistically to reduce tumor growth in vivo, which might have been due to enhancement of the immunotherapy efficacy by the anti-EMT effect of the anti-VEGF treatment [166].

## 6. EMT and Stemness

Mounting evidence [29,30,32,33,34,35,36] points to a small subset of cells within the tumor (cancer stem cells, CSCs) that can initiate tumors and drive metastasis and resistance to therapy. The American Association for Cancer Research defines a cancer stem cell as a tumor cell that can self-renew and generate the different lineages of differentiated cells composing the tumor [95].

The origin of a cancer stem cell remains debatable but two putative theories have been recently proposed: a normal stem cell could become an “activated stem cell” because of tissue damage or inflammation, or a mature differentiated cell could acquire the capacity of self-renewal to become an activated stem cell [167]. In a next step, the activated stem cell transforms into a cancer stem cell through mutations leading to inactivation of tumor suppressor genes and/or activation of oncogenes [167]. In addition, EMT is known as a driver of stemness [62].

Different mechanisms are responsible for the therapy resistance of CSCs. Like normal stem cells, they protect themselves against chemical mutagens, for example chemotherapy, by expressing drug efflux pumps such as ATP-binding cassette transporters [168]. Furthermore, they are more resistant to DNA damage because they inherently have greater DNA repair capacities [167]. In addition they proliferate slowly and express higher levels of anti-apoptotic genes [62,95].

With regard to ovarian cancer, several authors have shown that EMT induces stem-cell like cells [33,169] that are associated with therapy resistance [32,35]. For example, TGFβ1-induced EMT triggered splice isoform switching of CD44, with upregulation of the mesenchymal variant CD44s, which promoted CSC-like features [169]. Moreover, knock-down of Snail in OVCAR8 cells decreased the expression of CSC markers such as CD117, CD133, and Nanog, and increased sensitivity to platinum-based chemotherapy [32]. That the CSC-like population arises after only 3-5 days of chemotherapy, indicates that this population is probably already present in the chemonaive tumor and is selected by the chemotherapy [29].

Interestingly, it was recently reported that mechanical stress, e.g., shear stress due to intra-abdominal fluid flow, might influence the behavior of tumor cells. Shear stress induced the formation of CSCs (with upregulation of Oct-4, c-Kit, ABCG2, and P-glycoprotein) and resistance to cisplatin and paclitaxel in vitro, through downregulation of miR-199a-3p and subsequent activation of the PI3K/Akt pathway [170].

In summary, these findings point to cancer stem cells as drivers of therapy resistance in ovarian cancer. Therefore, successfully targeting these crucial cells might be pivotal for overcoming resistance to chemotherapy. The precise mechanisms by which CSCs evade current therapeutic agents need more research. However, the lack of reliable cell surface markers makes it difficult to enrich a pure CSC population.

## 7. Targeting EMT in Ovarian Cancer

The above-mentioned evidence indicates that EMT contributes to chemotherapy resistance and stemness in ovarian cancer, making it a potential target to tackle therapy resistance. Several authors have reported that anti-EMT therapies reverse therapy resistance in vitro or in vivo. One example is the potent TGFβ inhibitor, SB-431542, which increased carboplatin sensitivity [61]. Likewise, synthetic inhibitory antibodies to the type II TGF-β receptor (TGFBR2), reversed EMT and improved the response to carboplatin and anti-tumor immunity in vivo (fewer Tregs and more cytotoxic immune cells in the tumors) [38]. Furthermore, treatment with siRNA targeting TWIST increased platinum sensitivity in vivo. This siRNA was delivered into the cells by using hyaluronic-acid conjugated mesoporous silica nanoparticles (MSN-Has), which specifically bind to CSCs via their native ligand CD44 [171]. Similarly, treating a preclinical orthotopic mouse model with nanoliposome-delivered miR-15a and miR-16 improved cisplatin sensitivity [94]. These nanoparticles passively concentrated at the tumor site due to the leaky vasculature, which has more space between the endothelial cells compared to normal blood vessels [172]. Furthermore, treating SKOV-3/DDP cell lines with PD98059, a MEK inhibitor, led to silencing of the ERK-pathway and EMT, making the cells more sensitive to cisplatin [105]. Another example is ginsenoside Rb1 and its metabolite compound K, which sensitized ovarian cancer cells to cisplatin and paclitaxel by inhibiting Wnt/β-catenin signaling and EMT both in vivo and in vitro. The effect on therapy resistance was mediated by inactivation of the drug efflux transporters ABCG2 and P-glycoprotein [173]. Indeed, ABCG2 and other ABC drug transporters are known transcriptional targets of the β-catenin/TCF pathway [174]. Finally, luteolin (5,7,3′,4′-tetrahydroxyflavone), a flavonoid in fruits and vegetables, sensitized ovarian cancer cell lines to paclitaxel by inhibiting phosphorylation of FAK/ERK which leads to decreased nuclear expression of p65 and subsequently reduced EMT [175].

## 8. Controversy about the Role of EMT in Ovarian Cancer Progression and Therapy Resistance

Despite the overwhelming evidence for the contribution of EMT to ovarian cancer progression and therapy resistance, several publications have stated the opposite. For instance, among 46 ovarian cancer cell lines classified as epithelial or mesenchymal based on a previously published expression signature [176], the epithelial cell lines were more platinum-resistant than the mesenchymal ones [177]. These contradictory results sometimes raise the question whether EMT truly causes ovarian cancer progression and therapy resistance. Several confusing results might be explained by the fact that some authors specifically investigated end-stage EMT with a full-blown mesenchymal phenotype. However, cancer cells in partial EMT-states are probably more important for cancer progression and therapy resistance, again indicating the importance of cell plasticity [18]. Another problem is that the expression of EMT transcription factors is often increased only transiently during specific steps of cancer progression, which makes it difficult to detect [26]. For instance, to form a macroscopic metastasis, the reverse process of MET is required and EMT transcription factors are often downregulated [63]. Finally, the leading EMT transcription factors (Zeb1, Zeb2, Snail, Slug, and Twist1) are all capable of inducing (a form of) EMT, but additionally have separate non-redundant functions that are tissue- and context specific. In addition, they can influence each other’s expression, resulting in a complex regulatory network [26]. All things considered, investigation of EMT is extremely complex. Obviously, a better understanding of how EMT influences ovarian cancer progression and therapy resistance warrants more research.

## 9. Conclusions

Since 80% of patients with ovarian cancer relapse within five years after the initial response to therapy, the major hurdle to improving their prognosis is preventing development of therapy resistance. Growing evidence shows that EMT induces chemotherapy resistance and stemness. Here, we reviewed different mechanisms that lead to EMT-induced chemotherapy resistance. Several authors report promising results with preclinical anti-EMT therapies. However, more research is needed to elucidate the mechanisms leading to EMT-induced therapy resistance before anti-EMT therapies can be effectively tested in clinical trials.
